# Supplementary material for: Evaluation of the Effectiveness and Use of Anti-Methicillin-Resistant Staphylococcus aureus Agents for Aspiration Pneumonia in Older Patients Using a Nationwide Japanese Administrative Database
Source: Microorganisms. 2023 Jul 27;11(8):1905. doi: 10.3390/microorganisms11081905 (PMC10456764; doi:10.3390/microorganisms11081905)
Supplement: Supplementary file 1 [file microorganisms-11-01905-s001.zip › microorganisms-2504123-supplementary.pdf]

Supplementary Table S1. Baseline characteristics of patients treated with or without anti-MRSA drug with respiratory failure group (a) and without respiratory failure group (b) before and after adjusting for confounders using the propensity score weighting method

(a)

Baseline characteristics	Before adjustment			After adjustment		
**Variables**	**Anti-MRSA agents combination group *n* = 515**	**Non-anti-MRSA agents group *n* = 54583**	**p-value**	**Anti-MRSA agents combination group *n* = 515**	**Non-anti-MRSA agents group *n* = 54583**	**p-value**
Age	83.21 (±8.18)	85.61 (±7.61)	<0.0001	85.23 (±0.35)	85.58 (±0.03)	0.31
Sex	66.41% (342/515)	54.35% (29666/54583)	<0.0001	58.20%	54.46%	0.07
Emergency admission	98.64% (7/515)	98.70% (53873/54583)	0.9073	98.29%	98.71%	0.641

Emergency transport	75.53% (389/515)	64.00% (34874/54583)	<0.0001	64.74%	64.01%	0.739
Respiratory medicine	7.29% (40/515)	7.77% (3977/54583)	0.6761	5.87%	7.29%	0.089
Advanced treatment hospital	5.24% (27/515)	2.99% (1632/54583)	0.0029	3.00%	3.01%	0.999
Dementia	75.15% (128/515)	70.32% (16199/54583)	<0.0001	66.11%	70.37%	0.046
Coma	18.64% (96/515)	10.02% (5469/54583)	0.0152	11.09%	10.10%	0.368
COPD	6.21% (32/515)	8.85% (4830/54583)	0.0359	10.33%	8.83%	0.472
Asthma	2.52% (13/515)	4.61% (2517/54583)	0.0243	5.38%	4.59%	0.57
NTM	0.19% (1/515)	0.17% (208/54583)	0.4923	0.00%	0.37%	<0.0001
IP	1.55%	1.59%	0.9964	1.71%	1.59%	0.732

	(8/515)	(867/54583)				
Parkinson syndrome	7.57% (39/515)	6.21% (3338/54583)	0.17	0.16%	0.17%	0.922
Sequelae of cerebrovascular disease	20.39% (105/515)	20.40% (11133/54583)	0.9964	20.66%	20.39%	0.876
Maintenance dialysis	2.91% (15/515)	0.80% (436/54583)	<0.0001	0.98%	0.82%	0.541
Emergency dialysis	0.19% (1/515)	0.00% (2/54583)	<0.0001	0.00%	0.01%	0.441
Abscess of lung and mediastinum	0.39% (2/515)	0.18% (97/54583)	0.2613	0.61%	0.18%	0.132
Pyothorax	0.19% (1/515)	0.36% (197/54583)	0.5291	0.00%	0.36%	<0.0001
Achalasia	0.19% (1/515)	0.30% (166/54583)	0.6514	0.04%	0.30%	<0.0001

Malignant neoplasms	9.71% (50/515)	11.22% (6124/54583)	0.0118	12.34%	11.21%	0.485
Malignant neoplasms in the oral cavity	0.97% (5/515)	0.33% (179/54583)	0.0304	0.11%	0.33%	<0.0001
Malignant neoplasm of the esophagus	0.58% (3/515)	0.74% (404/54583)	0.6776	0.31%	0.74%	<0.0001
Additional reimbursement for ICT	88.16% (454/515)	92.24% (50350/54583)	0.0006	93.70%	92.20%	0.039
Additional reimbursement for AST	55.73% (287/515)	50.76% (27706/54583)	0.0248	50.83%	50.80%	0.99
Surgery	9.13% (47/515)	2.25% (1229/54583)	<0.0001	2.04	2.32	0.454
IPPV	16.89% (87/515)	3.68% (2006/54583)	<0.0001	2.63%	3.79%	<0.0001

NPPV	1.36% (7/515)	0.48% (262/54583)	0.0044	0.87%	0.50%	0.288
ICU admission	8.54% (44/515)	1.11% (607/54583)	<0.0001	1.08%	1.18%	0.787
Dopamine	5.24% (27/515)	1.36% (744/54583)	<0.0001	1.51%	1.40%	0.769
Noradrenaline	0.78% (4/515)	0.11% (59/54583)	<0.0001	0.21%	0.11%	0.507
Steroid	17.67% (91/515)	8.83% (4819/54583)	<0.0001	7.81%	8.91%	0.358
Insulin	13.20% (68/515)	7.17% (3916/54583)	<0.0001	6.85%	7.23%	0.689
Albumin	6.21% (32/515)	0.75% (409/54583)	<0.0001	0.99%	0.80%	0.098
Immunoglobulin	0.81% (4/515)	0.11% (88/54583)	0.0009	0.08%	0.12%	0.276
Anti-influenza virus drug	1.75% (9/515)	1.41% (770/54583)	0.5192	1.82%	1.41%	0.295

TPN	18.25% (94/515)	2.94% (1604/54583)	<0.0001	3.36%	3.08%	0.549
Nasal tube feeding	12.82% (66/515)	4.11% (2243/54583)	<0.0001	4.72%	4.19%	0.455
Gastrostomy nutrition	0.19% (1/515)	0.01% (8/54583)	0.0015	0.02%	0.02%	0.687
Dysphagia rehabilitation	10.49% (54/515)	13.01% (7103/54583)	0.0894	12.77%	12.99%	0.877
Penicillin	60.58% (312/515)	77.04% (42050/54583)	<0.0001	78.30	76.88	0.34
Macrolide	1.55% (8/515)	0.61% (334/54583)	0.0068	0.42%	0.62%	0.26
First-generation cephalosporin	1.17% (6/515)	0.69% (379/54583)	0.2018	0.71%	0.70%	0.975
Second-generation cephalosporin	2.52% (13/515)	1.80% (981/54583)	0.2173	1.81%	1.80%	0.975

Third-generation cephalosporin	14.37% (74/515)	18.07% (9862/54583)	0.0298	16.20%	18.03%	0.165
Fourth-generation cephalosporin	1.55% (8/515)	0.84% (459/54583)	0.0792	0.87%	0.85%	0.922
Carbapenem	35.73% (184/515)	5.83% (3181/54583)	<0.0001	6.32%	6.11%	0.63
Clindamycin	2.33% (12/515)	2.21% (1205/54583)	0.8507	2.72%	1.14%	0.095
New quinolone (IV)	2.33% (12/515)	1.43% (783/54583)	0.0898	2.72%	1.14%	0.095
New quinolone (PO)	0.78% (4/515)	0.98% (533/54583)	0.646	1.07%	0.97%	0.836
Minocycline	0.58% (3/515)	0.63% (346/54583)	0.8837	0.47%	0.63%	0.497
Metronidazole	0.19% (1/515)	0.14% (77/54583)	0.7497	0.22%	0.14%	0.528

(b)

Baseline characteristics	Before adjustment			After adjustment		
**Variables**	**Anti-MRSA agents combination group *n* = 111**	**Non-anti-MRSA agents group *n* = 26097**	**p-value**	**Anti-MRSA agents combination group *n* = 111**	**Non-anti-MRSA agents group *n* = 26097**	**p-value**
Age	82.17 (±7.41)	84.83 (±7.64)	0.003	84.11 (±0.83)	84.82 (±0.05)	0.391
Sex	76.58% (85/111)	57.94% (15121/26097)	<0.0001	60.38%	58.02%	0.701
Emergency admission	92.79% (103/111)	95.89% (25025/26097)	0.1011	96.08%	95.90%	0.927
Emergency transport	45.05% (50/111)	30.86% (8053/26097)	0.0012	70.31%	69.07%	0.789

Respiratory medicine	4.50% (5/111)	5.41% (1411/26097)	0.6748	6.12%	5.40%	0.751
Advanced treatment hospital	13.51% (15/111)	2.64% (690/26097)	<0.0001	2.61%	2.69%	0.898
Dementia	64.86% (72/111)	70.35% (18359/26097)	0.2069	73.66%	70.32%	0.224
Coma	10.81% (12/111)	6.28% (1640/26097)	0.0502	5.02%	6.31%	0.479
COPD	5.41% (6/111)	6.53% (1704/26097)	0.6323	15.61%	6.52%	0.141
Diabetes	0.90% (1/111)	0.56% (146/26097)	0.6308	0.07%	0.56%	<0.0001
CKD	18.02% (20/111)	16.44% (4291/26097)	0.6550	18.17%	16.45%	0.721
NTM	0.90% (1/111)	0.54% (140/26097)	0.6004	0.20%	0.54%	0.01
IP	0.90%	1.05%	0.8807	0.19%	1.05%	<0.0001

	(1/111)	(273/26097)				
Parkinson syndrome	8.11% (9/111)	7.25% (1893/26097)	0.7292	8.26%	7.25%	0.705
Sequelae of cerebrovascular disease	20.72% (23/111)	21.92% (5721/26097)	0.7601	20.72%	21.92%	0.744
Maintenance dialysis	2.70% (3/111)	0.75% (197/26097)	0.0186	0.15%	0.76%	<0.0001
Pyothorax	1.80% (2/111)	0.24% (63/26097)	0.001	0.08%	0.25%	<0.0001
Malignant neoplasms	9.01% (10/111)	12.68% (3310/26097)	0.2455	5.30%	12.67%	0.01
Malignant neoplasms in the oral cavity	1.55% (1/111)	0.52% (137/26097)	0.5850	0.49%	0.53%	0.885
Additional reimbursement for ICT	90.09% (100/111)	90.49% (23617/26097)	0.8840	94.83%	90.49%	0.045

Additional reimbursement for AST	51.35% (57/111)	46.67% (12180/26097)	0.3241	53.97%	46.69%	0.25
Surgery	5.41% (6/111)	2.25% (588/26097)	0.0260	2.16%	2.27%	0.948
ICU admission	0.90% (1/111)	0.01% (25/26097)	0.0072	0.13%	0.10%	0.652
Steroid	9.01% (10/111)	5.03% (25/26097)	0.0561	4.92%	5.05%	0.940
Insulin	11.71% (13/111)	5.67% (1480/26097)	0.0061	6.86%	7.23%	0.689
Albumin	0.90% (1/111)	1.95% (51/26097)	0.0956	0.01%	0.20%	<0.0001
Immunoglobulin	0.90% (1/111)	0.03% (7/26097)	<0.0001	0.01%	0.03%	0.033
Anti-influenza virus drug	1.80% (2/111)	0.61% (160/26097)	0.1108	0.97%	0.62%	0.262
TPN	6.31%	1.29%	<0.0001	1.37%	1.31%	0.924

	(7/111)	(336/26097)				
Nasal tube feeding	11.71% (13/111)	3.54% (925/26097)	<0.0001	3.31%	3.57%	0.813
Dysphagia rehabilitation	13.51% (15/111)	14.40% (3759/26097)	0.7897	14.23%	14.40%	0.959
Penicillin	56.76% (63/111)	71.65% (18698/26097)	0.0005	76.46%	71.58%	0.237
Macrolide	0.90% (1/111)	0.31% (81/26097)	0.2663	0.68%	0.31%	0.430
First-generation cephalosporin	1.80% (2/111)	1.57% (409/26097)	0.8427	3.32%	1.57%	0.372
Second-generation cephalosporin	2.70% (3/111)	2.66% (695/26097)	0.9794	1.19%	2.66%	0.045
Third-generation cephalosporin	16.22% (18/111)	22.22% (5800/26097)	0.1285	18.73%	22.20%	0.353

Fourth-generation cephalosporin	1.80% (2/111)	0.92% (240/26097)	0.3322	0.28%	0.92%	<0.0001
Carbapenem	32.43% (36/111)	2.92% (762/26097)	<0.0001	3.51%	3.05%	0.51
New quinolone (IV)	0.90% (1/111)	1.24% (323/26097)	0.7486	0.26%	1.24%	<0.0001
Minocycline	1.80% (2/111)	0.59% (154/26097)	0.0977	0.89%	0.60%	0.502
